# Design and synthesis of nano-biomaterials based on graphene and local delivery of cerebrolysin into the injured spinal cord of mice, promising neural restoration†

**DOI:** 10.1039/d3na00760j

**Published:** 2024-01-08

**Authors:** Ayda Yari-Ilkhchi, Mehrdad Mahkam, Abbas Ebrahimi-Kalan, Hamid Soltani Zangbar

**Affiliations:** a Chemistry Department, Faculty of Science, Azarbaijan Shahid Madani University Tabriz Iran 5375171379 mmahkam@yahoo.com mmahkam@gmail.com mahkam@azaruniv.ac.ir ayda.yari90@gmail.com; b Neuroscience Department, Faculty of Advanced Medical Science, Tabriz University of Medical Sciences Tabriz Iran

## Abstract

Spinal cord injury (SCI) is an incurable and catastrophic health issue with no clinical solution. As part of cascade reactions, the inflammatory process and fibrous glial scar production aggravate the amount of lesion through a secondary damage mechanism, encouraging scientists from other disciplines to investigate new paths for solving this problem. Graphene oxide (GO) and its derivatives are among the most promising biomedical and nerve tissue regeneration materials due to their remarkable chemical, mechanical, and electrical properties. This paper designs and introduces a new GO-based nanomaterial to minimize inflammation and stimulate neurite regrowth. To improve biocompatibility, biodegradability, and cell proliferation, GO plates were modified with polyethylene glycol (PEG) and Au nanoparticles as neuroprotective and antibacterial agents, respectively. Preliminary biological investigations on bone marrow derived mesenchymal stem cells (BM-MSCs) with various concentrations of a graphenic nanocarrier indicated a lack of cell toxicity and an enhancement in BM-MSC proliferation of about 10% after 48 hours. Therapeutic nanostructures were used in the T10 segment of a mouse SCI model. The pathological and immunohistochemical data revealed that refilling tissue cavities, decreasing degeneration, and establishing neuroregeneration resulted in a considerable improvement of hind limb motor function. Furthermore, compared to the nanocomposite mixture alone, the intraspinal delivery of cerebrolysin (CRL) had a more satisfying impact on nerve regrowth, cystic cavity, hemorrhage avoidance, and motor function enhancement. This study demonstrates the potential of graphenic nanomaterials for SCI treatment and neuroregeneration applications.

## Abstract 2

The preparation and application of injectable graphene oxide derivatives to the T10 segment of the SCI mouse model, coupled with the localized delivery of CRL into the lesion site, demonstrated a more profound impact on nerve regeneration.

## Graphical abstract

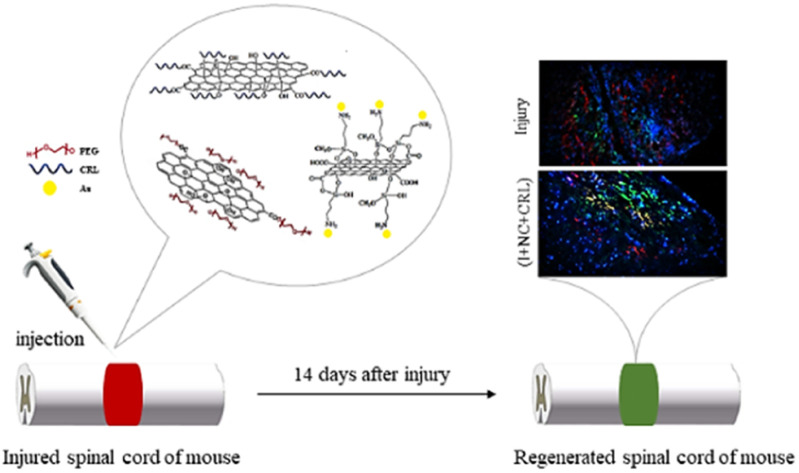

## 1. Introduction

SCI, a serious medical problem, is correlated with intrusive damage to sensory, motor, and voluntary actions at the injury site. Recovery and complete care of damaged nerves have been more challenging than for other tissues due to nervous system structure and function complexities. Progress has been made in understanding the degeneration mechanism and treatment methods. Still, almost three million people worldwide suffer from SCI, with tragic personal and social consequences and high financial costs (more than four billion dollars annually).^1–3^ Cascade interactions such as cystic cavitation, generation of glial scar, and the aggregation of inhibitors associated with myelin appear in the SCI lesion site microenvironment during the acute process.^4^ By completing the process of glial scar formation, functional nerve recovery leads to failure in nervous tissue regeneration and movement restoration.^5,6^ One challenge to efficient neurogenesis is how to rebuild the microenvironment for nerve tissue improvement. Functional studies and clinical trials are utilized to resolve these obstacles, including cell^7^ and biomaterial placement,^8^ growth factor release,^9^ inhibitory matrix disruption,^10,11^ endogenous neurite outgrowth, and axonal cytoskeleton stimulation.^6,12^ Using cells, biomolecules, biomaterials, or combinations thereof as effective methods can provide the least invasive form of SCI treatment. Biomaterial scaffolds can bridge the caudal and rostral sides of the spinal cord by filling the trauma gap, which holds great hope for neurite regeneration.^13–15^

Regenerative medicine, a brilliant field of medicine in recent decades, is an exciting method in which injured organs, tissues, or cells are renewed through special continuous strategies. Recently, regenerative medicine has been used to repair and regenerate bone, muscle, heart, nerve tissue, *etc.* In addition to stem cell therapy, biomaterials or a combination thereof have been used for neuroregeneration.^16^

Graphene-based nanomaterials (GBNs) are one of the most promising structures for nanotechnology applications due to their superior physicochemical properties. GBNs are used extensively in biomedical areas such as biomedicine,^17^ drug/gene delivery,^18^ neuroscience,^19^ bioimaging,^20^ phototherapy,^21^ biosensor fabrication,^22^ and tissue engineering.^23^ In addition to the significant characteristics of graphene (G), GO, and partially reduced GO (rGO), as well as other graphene derivatives with greater versatility and hydrophilicity for bio-applications,^24,25^ can enhance neural differentiation and axonal coordination through recording and stimulating neural electrical signals.^9,26^

The biocompatibility and toxicity of GBNs are currently a source of concern. Various parameters, including diameter, concentration, chemical compositions of the surface, lateral dimension, and aggregation conditions, decisively impact cytotoxicity.^27,28^ Proper functionalization of the GO surface is therefore essential for designing a nanocarrier system with high *in vivo* biocompatibility and biodegradability, good solubility in the body's physiological environment, and the development of antibacterial properties.

PEG is a synthetic polymer that is remarkably hydrophilic, biocompatible, and Food and Drug Administration (FDA) approved for grafting on GO sheets covalently and non-covalently. PEG modification could delay the circulation time and improve the *in vivo* pharmacokinetics for effective drug delivery. Furthermore, it has shown activities such as neuroprotection, anti-inflammatory properties, suppression of conversions in the SCI environment, and the ability to cross the blood–spinal cord barrier.^29,30^

Besides PEGylation, additional chemical functionalization should be examined to develop the antibacterial activities of GO materials. Subsequently, chitosan or metal antibacterial agents (silver nanoparticles (AgNPs), gold nanoparticles (AuNPs), zinc oxide (ZnO), and others) can be used. AuNPs have displayed superior performance in electronic, sensing, catalytic, and biomedical applications.^31–33^ In order to enhance conductivity and anti-inflammatory activity, the combination of AuNPs with GO can be an attractive approach to develop cell proliferation and nerve improvement.

On the other hand, local drugs, trophic factors, and cell delivery provide an exceptional opportunity to localize carriers at the injury site. Some neurotrophic factors have been employed in the cell therapy of SCI to ensure the survival of damaged cells and stimulate the growth of damaged axons.^13,34–36^

CRL, a low-molecular-weight peptide, is a porcine brain-driven preparation that contains a balanced composition of several neurotrophic factors to develop neuroprotection, neuroplasticity, and neuroregeneration in various brain diseases.^37^ It was used as a model medication to examine the manufactured smart nanocarrier's loading and release behavior and improve neural regeneration in SCI challenges.

**Fig. 1:**
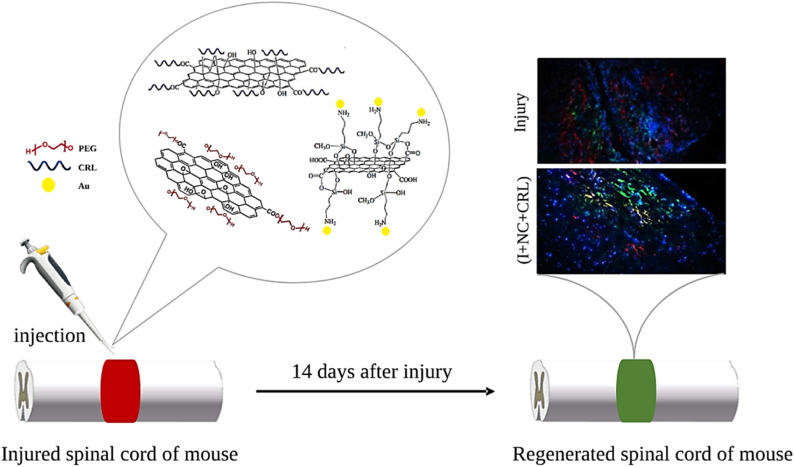
Schematic representation of the procedure.

This study aimed to assess the creation and usage of GO-based nano-biomaterials as a neuro-stimulator and drug carrier for SCI treatment. GO was chosen due to its excellent physicochemical qualities and ease of reaction. The GO surface was first modified with PEG and AuNPs to enhance biocompatibility, physiological solubility, antibacterial properties, and conductivity of GO to improve treatment quality and reduce regeneration time. Meanwhile, the encapsulation and release abilities of the CRL were evaluated using GO plates. The injectable nanostructures were employed in a mouse's damaged thoracic spinal cord after analyzing *in vitro* cell toxicity. The functional improvement was measured two weeks after surgery. Histological examinations were also performed to confirm the tissue response, neurite presence, and glial scar density in the lesion microenvironment (Fig. 1).

## 2. Experimental

### 2.1 Materials

Natural flake graphite, sodium nitrate (NaNO_3_), potassium permanganate (KMnO_4_), sulfuric acid (H_2_SO_4_, 98%), hydrochloric acid (HCl), hydrogen peroxide (H_2_O_2_, 30%), DMSO, sodium hydroxide (NaOH), ethanol 96%, 3-aminopropyltriethoxysilane (APTES), tetraethyl orthosilicate (TEOS), and ammonia were purchased from Merck. 3-(4,5-Dimethylthiazol-2-yl)-2,5-diphenyltetrazolium bromide (MTT), polyethylene glycol 400, 2′,7′-dichlorofluorescein diacetate (DCF-DH), and chloroauric acid (HAuCl_4_) were obtained from Sigma-Aldrich. Dulbecco's Modified Eagle Medium (DMEM), penicillin–streptomycin 100×, fetal bovine serum (FBS), phosphate-buffered saline (PBS), and trypsin–EDTA 0.25% were purchased from Gibco. Cerebrolysin was purchased from Ever Pharma.

### 2.2 Characterization apparatus

#### 2.2.1 Fourier transform infrared spectrometry (FTIR)

FTIR was used to investigate the structure of the synthesized nanobiocomposites (Bruker Vector-22 FT-IR spectrometer). The wavenumber (cm^−1^) *vs.* transmittance percent of each sample was measured in the 400 to 4000 cm^−1^ range, using a KBr pellet technique.

#### 2.2.2 X-ray diffraction analysis (XRD)

Powder X-ray diffractograms were obtained from dried nanomaterials employing CuKα radiation (*λ* = 1.542 Å), with the Bragg angle varying from 2 to 70° (Bruker AXS-D8 Advance diffractometer) to determine the chemical composition.

#### 2.2.3 Thermogravimetric analysis (TGA)

The Q500 (TGA Q500) analyzer was used to perform TGA. Nanomaterials were weighed and evaluated in a nitrogen environment. The temperature range was measured from 47 to 675 °C at a scanning rate of 10 °C min^−1^.

#### 2.2.4 Ultraviolet-visible spectrophotometry (UV-Vis)

UV-Vis spectra were performed with a Philips PU 8620 Ultraviolet spectrophotometer at the absorption maximum (*λ*_max_) using a 1 cm quartz cell.

#### 2.2.5 Field emission scanning electron microscopy (FESEM)

To determine the surface morphology and elemental composition of GBM, the samples were sputter-coated with a thin gold film. This coating makes them conductive for examination at 30 kV using a MIRA3 FESEM microscope equipped with energy-dispersive X-ray spectroscopy (EDX).

#### 2.2.6 Dynamic light scattering (DLS)

The charge of the surface, average dimension, and the polydispersity index (PDI) of the prepared nano-biomaterials were evaluated using a DLS (Microtrac, Nanotrac Wave) with a laser light wavelength of 780 nm at room temperature in deionized water (DW).

### 2.3 Preparation of GO–PG

As described previously, GO was prepared through the modified Hummers' procedure.^38,39^ Then, 100 mg of GO was dispersed in 100 mL of DW in a sonication bath for two hours. Then 10 mg of PEG was added to the GO suspension and stirred vigorously at 500 rpm and 60 °C for 24 h. The color changed from brown to black. The obtained solid was washed with DW five times for purification, centrifuged at 12 000 rpm for 5 minutes, and dried at 40 °C.

### 2.4 Preparation of GO–SiO_2_–NH_2_–AuNPs (GO–Au)

In brief, 100 mg of GO in 100 mL of ethanol : DW (20 : 80) was dispersed by ultrasonication for one hour to obtain one mg mL^−1^ solution. Then 0.5 mL of TEOS and 0.5 mL of APTES were inserted and redispersed for 30 minutes. The homogeneous solution was moved to the hotplate. After adding 100 μL ammonia, the mixture was softly stirred at 125 rpm and 40 °C for 12 hours and turned black. Then the amino-functionalized GO precipitate was washed with ethanol and distilled water to remove the unreacted chemicals. The obtained GO–SiO_2_–NH_2_ planes were redispersed in DW and mixed with HAuCl_4_·3H_2_O aqueous solution (2.5 mL, 0.01 M). Subsequently, the suspension was mixed with magnetic stirring at 96 °C for 30 minutes. Au nanoparticles were placed on the amine groups and the GO nanosheets. Finally, the prepared hybrids were filtered, washed with DW three times, and dried at 40 °C in a vacuum atmosphere.

### 2.5 Loading of CRL

70 mg of CRL solution in DW was added to 40 mL of GO aqueous dispersion (1 mg mL^−1^) and stirred at room temperature (20 °C) for 24 h. After centrifugation at 10 000 rpm, the solution was filtered. The GO–CRL was washed with DW to remove a small quantity of solubilized unbound drug and dried under vacuum at room temperature. The loading ratio of CRL (wt%) was derived from UV absorbance at 268 nm and calculated using the equations below.Entrapment efficiency (%) = the initial amount of drug − the amount of drug in the supernatant/the initial amount of drug × 100Loading efficiency (%) = the initial amount of drug − the amount of drug in the supernatant/amount of nanocarrier × 100

### 2.6 CRL release studies

GO–CRL (10 mg) in 5 mL of phosphate-buffered solution (PBS; pH 7.4) was placed in a dialysis bag (14 kDa MWCO) and transferred to a 10 mL buffer solution for 14 days at 37 °C. At predefined intervals (1, 2, 3, …, 14 days), the supernatant was collected and replaced with an equal amount of fresh buffer. The amount of CRL released was measured using a UV-Vis spectrophotometer and calculated using the equation below.Drug release (%) = ∑(amount of drug in release solution at time *t*)/amount of drug loaded in the nanocarrier × 100

### 2.7 Isolation of BM-MSCs and culturing

As explained previously, BM-MSCs were extracted from the tibia and femur bones of mice. The animal's whole body was dissected after immersing in 70% (v/v) ethanol. Applying micro dissecting scissors and a surgical scalpel, muscles, ligaments, and tendons were carefully separated from the bones. After transferring into ice-cold sterile PBS, the ends of the marrow cavity were cleaned with medium until the bones turned white before centrifugation at 2500 rpm for 5 minutes. The supernatant was removed, and a 5 mL total medium (containing DMEM, 10% FBS, and 100 U per mL penicillin–streptomycin) was added before transferring the suspension to a cell culture flask. This flask was then placed within a humidified environment maintained at 37 °C, with a 5% CO_2_ concentration, in order to attain a cellular confluence ranging from 70% to 90%. Following the 3^rd^ passage and elimination of macrophages, lipids, and blood cells, the BM-MSCs were characterized employing CD44, CD34, and CD90 surface markers using the flow cytometry technique in the previous study.^39^

Each specimen underwent a thorough cleansing process employing 70% ethanol, followed by desiccation within a sterile hood. Subsequently, a 30 minute UV irradiation sterilization procedure was administered prior to their utilization in both *in vitro* and *in vivo* investigations. Notably, these assessments were conducted on a minimum of three separate occasions.

### 2.8 MTT assay

The BM-MSCs at 70% confluency were detached by trypsin and then centrifuged at 1000 rpm for 5 minutes at room temperature. The cells were seeded in 96-well plates with 200 μL complete DMEM medium and incubated overnight to adhere to the dishes. After removing the old media, the cells were treated with various quantities of GO, GO–PG, GO–Au, GO–CRL, and NC + CRL (0.1, 1, 10, 20, 40, 100, and 150 μg mL^−1^) and placed in an incubator set at 37 °C for durations of 24, 48, and 72 hours. The 96 wells were rinsed with PBS and incubated for 4 hours in darkness with 100 μL MTT in the culture medium (0.4 mg mL^−1^ medium) at 37 °C. The NC + CRL group is symbolized as a complex of GO–PG, GO–Au, and GO–CRL 1 : 1 : 1, used in the animal model. After discarding the MTT-containing medium, 200 μL of DMSO was added to dissolve the insoluble violet formazan produced by live cells. Ultimately, the spectrophotometric microplate ELISA reader (Multiskan MK3, Thermo Electron Corporation) was employed to measure the absorbance of the formazan solution at a wavelength of 570 nm, and the results were represented as optical density (OD) after blank subtraction.Cell viability (%) = OD (test)/OD (control) × 100

### 2.9 Reactive oxygen species (ROS) studies

Intracellular reactive oxygen species or radicals (ROS) were measured using 2′,7′-dichlorodihydrofluorescein diacetate (DCFH-DA, Sigma-Aldrich). First, 5 × 10^4^ cells were placed per well onto chamber slides and cultivated for 24 hours. The cell medium was then removed, and the cells were treated for 48 hours with GO, GO–PG, GO–Au, GO–CRL, and NC + CRL (the control cells received no therapeutic nanomaterials). After rinsing the cells twice with PBS, 10 μM per L DCFH-DA was applied to the cells, and they were located in a dark incubator at 37 °C for 30 minutes. In order to remove uncombined probes, cells were washed twice with PBS and treated with DAPI for 30 minutes in a dark environment before rinsing with PBS and then examined under a fluorescence microscope (Nikon E1000M, Japan).

### 2.10 SCI modeling in mice

The animal experiments and procedures were carried out in compliance with the Care and Use Guidelines of the Advanced Medical Sciences Faculty of the Tabriz University of Medical Science and were authorized (214/D/21085) by the Azarbaijan Shahid Madani University's Institutional Animal Ethics and Use Committee. All of the BALB/c mice (25–30 g) utilized in this study were kept under standard laboratory conditions with 22–23 °C, 50–60% humidity, 12/12 dark/light cycles, and free access to food and water.

Mice (3 males + 1 female)^40^ were randomly divided into three triplicate groups (*n* = 12 per group), including the injury (I) group (SCI with no injection), the (I + NC) group (SCI with a complex of GO–PG and GO–Au 1 : 1 in 100 μg per mL injection), and the (I + NC + CRL) group (SCI with a complex of GO–PG, GO–Au, and GO–CRL 1 : 1 : 1 in 100 μg per mL injection). The NC and CRL symbols were used instead of (GO–PG and GO–Au) and GO–CRL, respectively, to simplify the characters. All mice were anesthetized with ketamine and xylazine (80 : 20 mg kg^−1^) intraperitoneally before the surgery. A longitudinal incision was made along the lower thoracic vertebrae to expose the spinal column, and a laminectomy was performed at the T10 segment. The compression approach resulted in the injury (0.3 mm, 15 s), and 50 μL of the therapeutic nanomaterials corresponding to each group were injected into the lesion space. After the procedure, the incision was sutured in layers and the creatures were situated in a separate enclosure under a warm lamp for 0.5–1 hour before being transferred to their home cages. Furthermore, mouse bladders were manually emptied twice a day until the reflex of the urine system returned. They received a constant dose of ciprofloxacin (2 mg kg^−1^) and sterile normal saline for 5 days.

### 2.11 Locomotor function test

The Basso, Beattie, and Bresnahan (BBB) test was applied to measure the locomotion activity of the hind limbs following SCI. The BBB test involves a 21-point locomotor assessment scale administered for 4 minutes every day for two weeks by two unrelated observers.

### 2.12 Histological and immunofluorescence studies

At two weeks post-injury, the mice were put into deep anesthesia with intraperitoneal injections of xylazine–ketamine (20 : 80 mg kg^−1^). Then they were sacrificed utilizing the usual perfusion-fixation method, which included sterile normal saline and paraformaldehyde 4% circulation. A 1 cm spinal cord slice was taken *via* a dorsal laminectomy and submerged in 4% paraformaldehyde at 4 °C. Fixed tissues were placed horizontally in paraffin and cut into 5 μm sections with a microtome. After transferring the segments to the slides, they were stained with hematoxylin and eosin (H&E) stains and studied using an optical microscope (Nikon Eclipse E100).

Immunofluorescence studies were performed for glial fibrillary acidic protein (GFAP; 1 : 500, Sigma Chemical Co.) and neurofilament (NF; 1 : 500, Sigma Chemical Co.). Following staining and washing with PBS, all sections were treated with rabbit/mouse FITC (1 : 50, Sigma Chemical Co.) and examined using a fluorescence microscope.

### 2.13 Statistical analysis

Data were expressed as the mean ± SD of at least three different experiments (*n* ≥ 3). GraphPad Prism (8.0.2) and ImageJ Fiji were utilized for quantitative analysis. The means of the data sources were calculated using the Student's *t*-test and one-way ANOVA. All statistical analyses determined the significance value as *P* < 0.05.

## 3. Results and discussion

### 3.1 FT-IR

Fig. S1† shows the FT-IR spectra of GO, GO–SiO_2_–NH_2_, GO–Au, PEG, and GO–PG. According to Fig. S1B,† the peaks at 1133 cm^−1^ and 1624 cm^−1^ are due to Si–O–C and C–N bonds. The absorption bands at 3360 cm^−1^ and 3400 cm^−1^ represent the primary aliphatic amine (N–H) groups, while the peak at 3400 cm^−1^ overlaps with the hydroxyl groups of GO. The peaks associated with C–H and C=C stretching vibrations of GO appeared at 2925, 2962, and 1635 cm^−1^ respectively. Additionally, the peak at 1068 cm^−1^ that corresponds to Si–O–Si bonds overlaps with that of C–O–C at 1065 cm^−1^, which indicates the successful functionalization of GO with TEOS and APTES.^41,42^ The GO–Au spectrum should be comparable to the GO–SiO_2_–NH_2_ peaks owing to the emergence of Au peaks in the far-infrared, but a new peak at 462 cm^−1^ might be possible and be attributable to the presence of Au nanoparticles.^43^ Further analytical procedures, such as XRD, were utilized to confirm the presence of AuNPs on GO sheets. In the spectrum of GO–PG (Fig. S1E†), the peaks of C=O and C–O became sharper and shifted to 1736 cm^−1^ and 1080 cm^−1^ respectively, because of the interactions between the GO's carboxylic acid and hydroxyl groups with O–H groups of PEG. The existence of peaks at 2877 cm^−1^ and 1456 cm^−1^ attributed to the –CH_2_ (sp^3^) and CH plane bending groups of PG and also the peak at 1635 cm^−1^ (C=C) of GO indicated grafting of PEG onto GO–PG.^44^

### 3.2 XRD

The crystalline structures of GO, GO–PG, GO–SiO_2_–NH_2_, and GO–Au have been investigated by XRD diffraction patterns. As shown in the diffractograms (Fig. S2†), the GO–PG diffractogram exhibits a broad peak at 2*θ* = 21.3° (120), slightly different from the GO–PEG pattern in the previous study,^39^ indicating a reduction in the crystallinity and development of an amorphous system with an interlayer spacing of *d* = 9.1 Å which may be due to weak interactions or a low degree of PEGylation. The broader and shorter diffraction pattern of GO–SiO_2_–NH_2_ at 2*θ* = 22.4° with an interplanar distance *d* = 3.96 Å compared to the GO pattern displays the silane functionalization of GO. The GO–Au pattern, besides the extensive GO–SiO_2_–NH_2_ peak, contains five sharp peaks at diffraction angles of 38.28°, 44.48°, 64.6°, 77.44°, and 81.58°, corresponding to the characteristic gold crystalline sheets (111), (200), (220), (311), and (222) respectively (ICDD code 00-002-1095).^45^

It should be noted that in diffraction patterns, the characteristic GO peak has vanished because of complete exfoliation and the existence of oxygen-containing groups. Moreover, the addition of PEG, TEOS, and APTES prevents aggregation and re-stacking of GO sheets. The presence of a broad peak observed in the diffractograms of GO–PG and GO–SiO_2_–NH_2_ near the natural graphite angle can be attributed to a partial reduction of graphene oxide (GO) induced by grafting reactions.

### 3.3 TGA/DTG

As shown in the TGA/DTG diagrams (Fig. S3a and b†), the weight loss near 100 °C is due to the evaporation of water molecules between layers. Almost 20% of the GO–PG mass decreases at 150–250 °C and 25% declines close to 250–350 °C, respectively, owing to the loss of groups of oxygen and PEG. The weight loss between 300 and 600 °C is attributed to the pyrolysis of carbon skeletal structures and the release of carbon dioxide into the atmosphere.^46^ The GO–SiO_2_–NH_2_ and GO–Au patterns display tremendous thermal stability against GO, forming a silica layer and gold nanoparticles on the GO surface.^40^ Two negligible weight reductions in areas less than 100 °C and 170–250 °C suggest the presence of moisture and oxygen groups, respectively. Due to silyl group decomposition, almost 25% of weight loss occurred between 250 and 350 °C. The other declination level near 420–450 °C illustrates the degradation of inert Au nanoparticles. As can be seen, the composites' thermal stability was significantly increased due to the successful surface modification of GO.^45,47^

### 3.4 FESEM and EDX

**Fig. 2:**
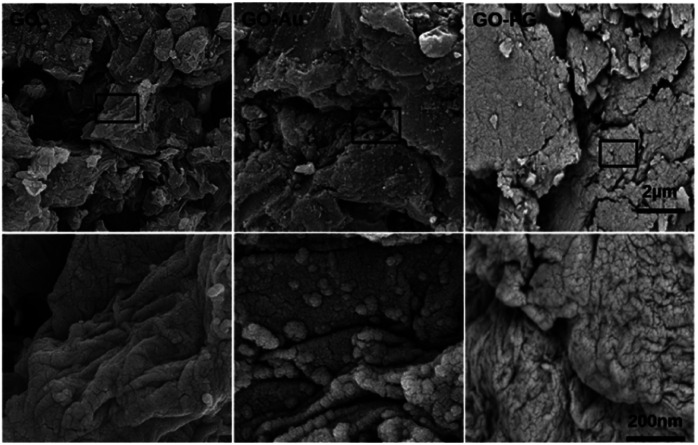
FESEM images of GO, GO–PG, and GO–Au with a scale bar of 2 μm and 200 nm.

FESEM images in Fig. 2 illustrate GO, GO–PG, and GO–Au nanocomposite morphological structure. GO sheets exhibit a planar structure with wrinkled surfaces. GO–PG depicts an extensive flat system, owing to H-bonding interactions between PEG and GO surfaces. In the figure of GO–Au, the white stains on the surface of GO related to the existence of silyl groups and Au nanoparticles are distributed uniformly on the GO surface.

Meanwhile, to record the confirmation of the elemental combination of the GO–PG and GO–Au nanocomposites, EDX was employed (Table 1). The elemental analysis data demonstrated that the modification reactions on the GO sheets were executed successfully.

**Table 1:** Elemental analysis by EDX spectrometry (wt%)

	C	O	N	Si	Au
G	100				
GO	56.4	43.6			
GO–PG	57.2	42.8			
GO–Au	37.4	36.7	14.3	7.2	4.4

### 3.5 DLS

In order to measure the size of nano-biocomposites, we have used dynamic light scattering (DLS) in water at room temperature. The suspended sizes of GO, GO–Au, and GO–PG were 170 nm, 226 nm, and 427 nm, respectively. Zeta potential and polydispersity analyses (Table 2) of the prepared nanomaterials showed that the PG and Au could increase the zeta potential of GO sheets by contributing negative charges to their surface, making them more stable and less prone to agglomeration in DW at room temperature. In addition, it could be noted that negatively charged nanomaterials produced can enhance cell attachment, proliferation, and differentiation through electrostatic attraction with cell surface components.

**Table 2:** DLS analysis of GO, GO–Au, and GO–PG

	GO	GO–Au	GO–PG
Mean size (nm)	∼170	∼226	∼427
Zeta potential (mV)	−44	−38	−57
PDI	0.63	0.28	0.18

### 3.6 UV-Vis spectra

**Fig. 3:**
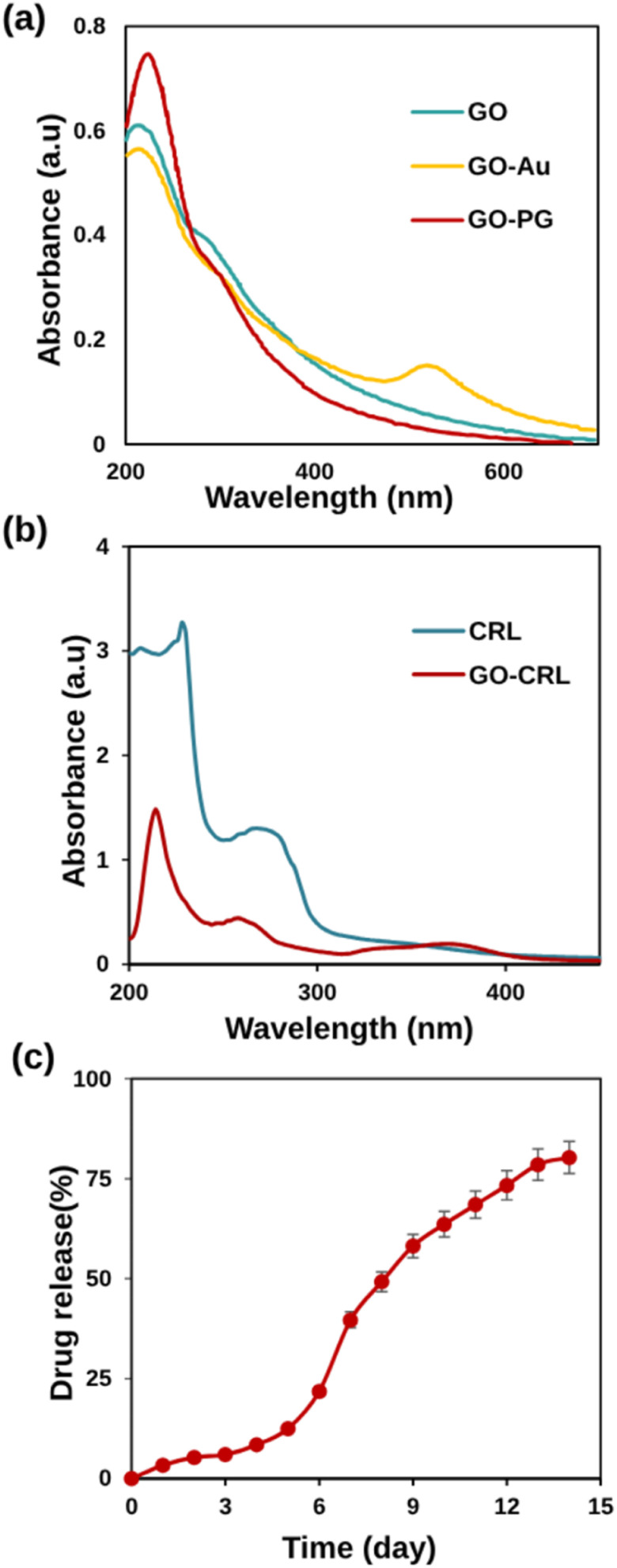
(a) UV-Vis absorption spectra of GO, GO–PG, and GO–Au in DW, (b) UV-Vis spectra of CRL and GO–CRL, (c) drug release percentages against time (day) in physiological pH (7.4) and 37 ± 1 °C.

UV-Vis absorption spectra, an effective method for characterizing grafted AuNPs on GO sheets, are demonstrated in Fig. 3a. At 230 and 300 nm, the absorption peaks correspond to π → π* (C=C) and n → π* (C=O) excitation in the GO curve. The n → π* peak shows a bit of red shift after Au and PEG modification, owing to GO partial reduction. Meanwhile, a new peak at 525 nm has emerged in the GO–Au curve, indicating the presence of Au nanoparticles in GO sheets.^48^ The spectrum of GO–PG was identical to that of GO, except for a greater absorbance, which may be attributed to non-covalent PEGylation.

### 3.7 *In vitro* drug loading and release evaluation

In this research, non-covalent drug loading was carried out at room temperature overnight. The presence of carboxylic acid groups on GO electrostatically interacts with CRL's amine groups, causing increased loading. Fig. 3b shows that CRL molecules were successfully loaded onto GO sheets, as evidenced by the characteristic absorption peak of CRL (268 nm) and GO–CRL. The loading concentration of CRL (92%) and the entrapment efficiency (96.5%) were determined using UV-Vis spectrophotometry. The *in vitro* release tests were carried out for 14 days, employing PBS at a temperature of 37 °C. According to the findings (Fig. 3c), the low quantity of CRL released from the nanocarrier (39.68% in the first week) increases to 80.27% after 14 days. This phenomenon could be attributed to the removal of the adsorbed drug from the nanocarrier as well as the solid electrostatic interaction between the CRL's amine groups and the nanocarrier's carboxylic acid groups. The calibration standard curve of CRL is shown in Fig. S4.†

### 3.8 MTT study

**Fig. 4:**
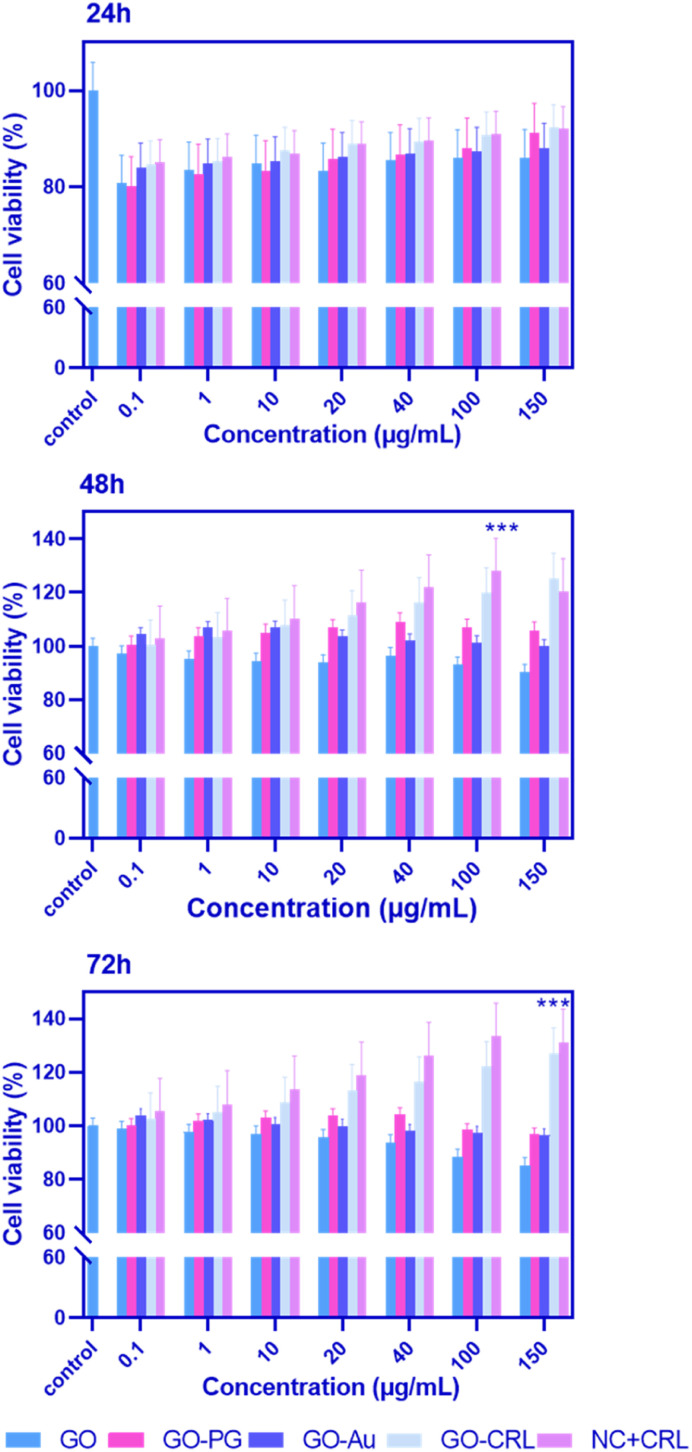
MTT assay was used to determine the cytotoxicity and biocompatibility of different GO–PG, GO–Au, GO–CRL, and NC + CRL doses on BM-MSCs after 24, 48, and 72 hours. The *p*-value for a significant difference between NC + CRL and control groups was 0.0001 (*n* = 3, mean + SD).

For biomedical applications, ideal nanomaterials must be biocompatible. A standard MTT assay was used to examine the *in vitro* cytotoxicity of the produced nano-biomaterials on BM-MSCs. Fig. 4 demonstrates the cytotoxicity results with a series of doses (0.1, 1, 10, 20, 40, 100, and 150 μg mL^−1^) of GO–PG, GO–Au, GO–CRL, and NC + CRL in a complete medium for 24, 48, and 72 hours. According to the findings, the cell viability of entire nanomaterials was greater than 80% except in high doses of GO–Au (100 and 150 μg mL^−1^). It can be deduced that no noticeable cytotoxicity was observed in the prepared nanocomposites after 24 hours. Cell proliferation for GO increased to more than 92% after 48 and 72 hours of exposure due to covalent and non-covalent interplays between hydrophilic GO and medium proteins. Here, through the activation of the GO surface, a noticeable enhancement in cell proliferation was achieved, reaching approximately 106% and 115% improvement when GO plates were grafted with both Au and PEG. In addition, due to the low PEGylation degree of GO, GO–PG increased cell growth and proliferation by around 5% compared to our earlier study. Regarding the GO–CRL and NC + CRL groups, cell viability increased to almost 120% and 133% in 72 hours, showing more effective interactions even with the small amount of CRL. Using GraphPad Prism, the effective concentration (EC_50_) values of GO, GO–PG, and GO–Au were 48, 53, and 10 μg mL^−1^, respectively. Numerous previous investigations have shown that dimension, concentration, chemistry of the surface, and shape may impact the cytotoxicity of products based on graphene.^28,49–51^

### 3.9 ROS study

**Fig. 5:**
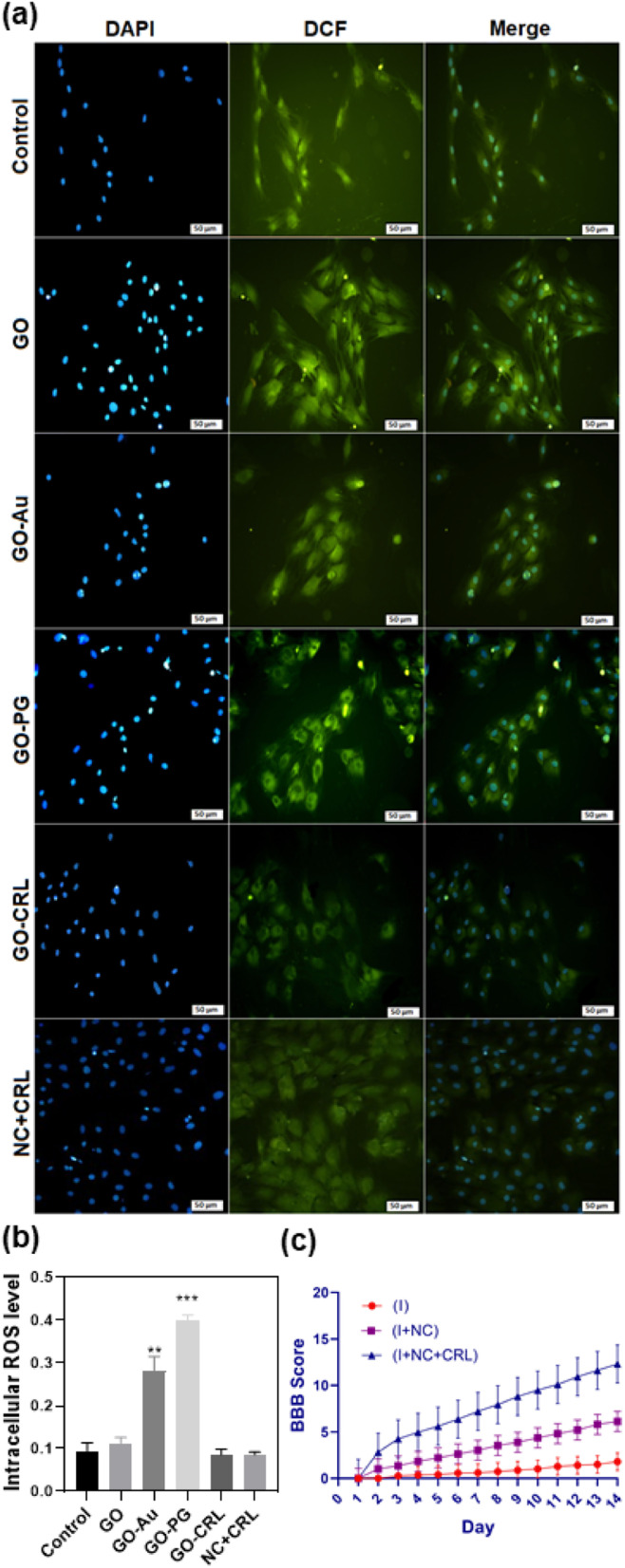
ROS generation in BM-MSCs after incubation with GO, GO–Au, GO–PG, GO–CRL, and NC + CRL for 48 hours. DCFH-DA and DAPI were used to detect ROS and cell nuclei, respectively. (a) Fluorescence microscope images with a scale bar of 50 μm, (b) quantitative ROS levels of nanocomposites (**p < 0.001, ***p < 0.0001). (c) Results of motor function evaluation of mice hind limbs using the BBB scale 1–14 days after injury. Data are expressed as mean ± SD (****p < 0.0001, determined by one-way ANOVA).

Intracellular ROS levels might be increased by exposing cells to nanomaterials, which is the primary feature of intracellular oxidative stress.^52^ ROS can cause intracellular lipid peroxidation, protein inactivation (*via* nitration and oxidation), mitochondrial malfunction, and apoptosis or necrosis. DCFH-DA is one of the most widely utilized methods for directly assessing a cell's redox status compared to alternative ROS detection techniques.^53^ In this system, H_2_DCFDA, a non-fluorescent lipid-permeable compound, is transformed into 2′,7′-dichlorofluorescein (DCF) (green fluorescence) by an intracellular esterase reaction. Previous research has shown that graphene-based materials cause the formation of intracellular ROS in a time-, dose-, lateral dimension-, and surface-dependent manner, with concentration and average lateral size playing an essential role in oxidative stress.^53–55^ As shown in Fig. 5a and b, the levels of ROS after 48 hours for GO, GO–Au, GO–PG, GO–CRL, and NC + CRL, respectively, were 0.093 ± 0.011, 0.28 ± 0.027, 0.37 ± 0.010, 0.086 ± 0.011, and 0.088 ± 0.005, which is considered a good score compared to prior studies. In this regard, Chen *et al.* have indicated that modification of the GO surface with Au increases the level of ROS by around 4.7.^56^ These findings may be attributed to the nanomaterials' acceptable average lateral size and fewer edges. Furthermore, the combination of the nanomaterials in the NC + CRL group caused a desirable reduction in ROS levels due to both the pharmacological effect of CRL and covalent and non-covalent interactions among its functional groups with the oxygenated groups of GO–PG and GO–Au.

Meanwhile, the nuclei in the core of cells stained with DAPI (blue) showed a round morphology with no fragmentation. These results correlate with the MTT assay, indicating that the prepared nanomaterials are not only less toxic but also more biocompatible after 48 and 72 hours. In future studies, more detailed cellular investigations will be examined (Fig. 5a).

### 3.10 BBB tests

Recovery of locomotor function was assessed with BBB score for injured animals at the T10 level for 4 minutes during the initial 2 weeks past injury (subacute phase). The locomotion behavior of animals in all groups was exactly equivalent immediately after injury, indicating a lack of motor function (Fig. 5c). Then, in comparison with the (I) group (1.8 points), the motor function in the (I + NC) and (I + NC + CRL) groups was restored astonishingly, with higher BBB ratings (6.12 and 12.32 points, respectively). Besides that, with continuously increased drug release into spinal cord lesion sites, the BBB score of the (I + NC + CRL) group was considerably higher than that of the other groups 7 days following surgery, demonstrating that (I + NC + CRL) had a superior therapeutic influence on functional improvement.

### 3.11 Histology and immunohistochemistry studies

Encouraged by the exceptional capabilities of graphenic nano-biomaterials and their potential to repair neurites, we investigated the tissue response of the damaged mouse spinal cord to a complex of GO–PG, GO–Au, and local delivery of CRL into the wounded location in the subacute phase.

**Fig. 6:**
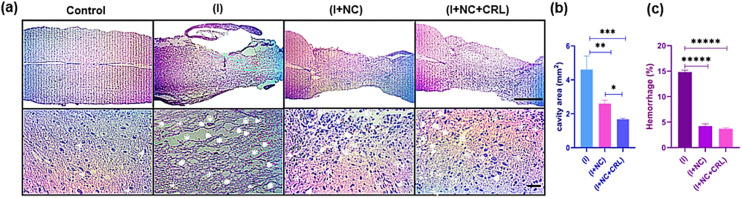
H&E staining was used to examine the histology of nerve tissue regeneration two weeks after surgery. Images from an optical microscope are exhibited at scales of 1 mm and 50 μm. (a) In the groups, the magnified pictures revealed cystic cavities (asterisks), bleeding (arrowheads), astrocytes (thick arrows), lymphocytes (thin arrows), and myelinated axons (thin arrowheads). Quantitative data for cavity areas and bleeding percent at longitudinal section lesion sites in the spinal cord (b and c). Mean ± SD, **p = 0.0031, ***p = 0.00045, and *p = 0.015 for cavity area and *p* ≤ 0.000001 for hemorrhage percent, *n* = 12 per group.

According to the mouse spinal cord diameter, we selected 50 μL of mixed nanomaterials to place into the damaged site and release CRL locally. Further studies will be performed in the chronic phase (over a longer period of time) in the mouse model. The H&E stains of sagittal sections indicated the standard histopathology of the control (without injury), (I), (I + NC), and (I + NC + CRL) groups two weeks after surgery in Fig. 6a. Numerous neuronal cells were observed in the control group, and myelinated axons were shown with thin arrowheads. Multiple cystic cavities (asterisks), hemorrhage (arrowheads), and round-shaped nucleus cells, most probably activated astrocytes (thick arrows), were seen surrounding the lesion site in the (I) group. The cavity area and hemorrhage percent were calculated using Fiji ImageJ. The cavity area in the (I) group was 5 ± 1.263 mm^2^, which was reduced to 2.5 ± 0.2 mm^2^ and 1.7 ± 0.131 mm^2^ in the (I + NC) and (I + NC + CRL) groups, respectively, indicating less visible and smaller-sized cavitation (Fig. 6b). The bleeding rate was also decreased from 14.5% ± 0.592 (I) to 3.25% ± 0.424 (I + NC) and 3.27% ± 0.430 (I + NC + CRL) (Fig. 6c). Moreover, a band of tightly compacted cells with oval nuclei could be identified at the rostral, medial, and caudal hemispheres, which most likely represented lymphocytes.

**Fig. 7:**
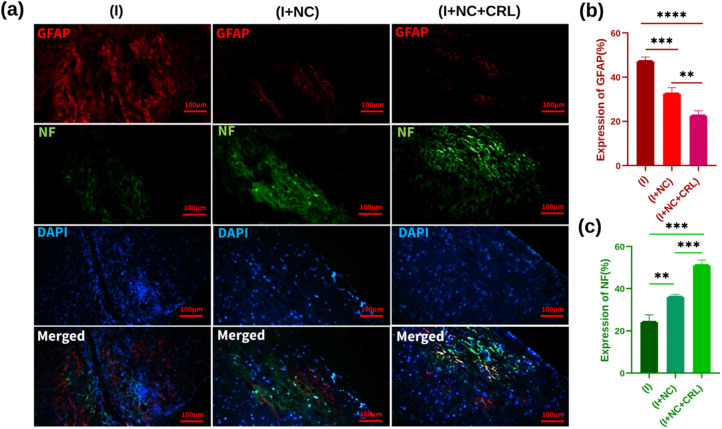
(a) Immunofluorescence staining of the sagittal sections of spinal cord tissue two weeks past operation. Images from a fluorescence microscope are exhibited at scales of 100 μm. (b) Quantitative data for GFAP staining with significant difference of **p = 0.0032, ***p = 0.0006, and ****p ≤ 0.00001. (c) Quantitative data for NF staining with significant difference of **p = 0.0021, ***p = 0.0002. Mean ± SD, *n* = 12 per group.

To evaluate the effect of synthetic nano-biomaterials on neurite regeneration, we used immunofluorescence staining for GFAP (red), NF (green), and DAPI (blue) by double labeling. For nucleus staining, DAPI was utilized (Fig. 7a). GFAP, a marker of reactive astrocyte detection and glial scar formation, hindered neuroregeneration in injured areas. Fig. 7b demonstrates that GFAP was expressed substantially in the (I) group, near the lesion location, at around 48% ± 1.431. Treatments with the (I + NC) and (I + NC + CRL) significantly lowered GFAP accumulation to 33% ± 1.756 and 23% ± 1.652, respectively, indicating that CRL affects astrogliosis in comparison with (I + NC) and (I) groups. The NF immunofluorescence labeling was then utilized to trace and detect nerve fiber renewal and axonal regeneration. NF-positive nerve fibers were evident in the rostral, central, and caudal portions of the (I + NC) and (I + NC + CRL) groups as compared to the (I) group. The percentages of NF expression were calculated as 25% ± 2.847, 37% ± 0.545, and 52% ± 1.907 for the (I), (I + NC), and (I + NC + CRL) groups, respectively. For the group (I + NC + CRL), particularly extensive lengths of nerve fibers were seen in the center of the lesion. Fig. 7c depicts a significant difference in the quantitative analysis of NF expression.

These data show that compared to group (I), treatment groups (I + NC) and (I + NC + CRL) significantly reduced glial scar formation, cavity areas, bleeding, and increased neurite counts. In addition, local delivery of CRL at the lesion site accelerated the improvement process two weeks after the injury.

Previous studies have indicated that GBMs can repair SCI and promote nerve regrowth.^57–60^ Topography and surface chemistry of tissue engineering biomaterials have essential roles in influencing cell behaviors (differentiation, proliferation, directed growth of nerve cells, fibrosis, and inflammation) and regulating intracellular signaling pathways to affect gene expression. Zhang and coworkers found that surface modification of PLLA-aligned nanofibers with GO nanosheets enhances surface roughness, resulting in optimal contact for cell growth.^61^ Encapsulating several neurotrophic factors into biomaterials increases cell growth, proliferation, differentiation, migration, and neurite regeneration. However, nerve development is greatly influenced by the amount, duration, and location of neurotrophic substances released *in vivo*.^62^

In this study, we found that injecting graphene-based nano-biomaterials into the lesion cavity filled the damaged hole and reduced astrocyte hyperactivities for glial scar formation, hemorrhage, edema, and necrosis. On the other hand, the electrical ability of reduced GO sheets increases connectivity and stimulates neurite sprouting and outgrowth. Furthermore, smart delivery of CRL (I + NC + CRL group) into the damaged microenvironment 7 days after injury (concurrent with nerve stimulation) enhances locomotor hindlimb activity and accelerates neuroregeneration more effectively than the (I + NC) group.

## 4. Conclusions

This paper reports the creation of new biocompatible GO-based nanomaterials for nerve tissue engineering and neuroregeneration. Nanocomposites were functionalized with PEG and Au nanoparticles to improve conductivity, antibacterial, and neuroprotective characteristics, resulting in an optimal microenvironment for neurite regeneration. The abundance of oxygen-containing groups on the nanocomposites' surface establishes a constructive interplay with the proteins of the medium. This interaction not only fosters the proliferation of BM-MSCs but also maintains an optimal level of reactive oxygen species (ROS), which is highly favorable. Two weeks after injection of a mixture of prepared nano-biomaterials into the mouse spinal cord lesion site, a cascade of improvements became evident. Notably, there was an increase in functional locomotor scoring of the BBB. This positive trend was coupled with a reduction in cavity dimensions, instances of bleeding, astrocyte activation, and scar formation. Moreover, a marked enhancement in both reconnection and the regeneration of neurites was prominently observed. Local distribution of CRL significantly enhanced these results and SCI improvement. We highly suggest electrical stimulation of these materials, combined with cells and growth factors, to increase neural tissue repair and hindlimb functional recovery, providing a potentially curative approach for CNS disorders.

## Author contributions

AY-I designed, performed experiments, investigated the data, and wrote the article. MM designed, supervised and oversaw the entire project. AE-K supervised the cell culture and animal model, and HSZ helped with the animal model. All authors reviewed and commented on the manuscript.

## Conflicts of interest

The authors have no financial conflict to declare.

## Supplementary Material

NA-006-D3NA00760J-s001
