# Supplementary material for: Design and synthesis of nano-biomaterials based on graphene and local delivery of cerebrolysin into the injured spinal cord of mice, promising neural restoration
Source: Nanoscale Adv. 2024 Jan 8;6(3):990–1000. doi: 10.1039/d3na00760j (PMC10825937; doi:10.1039/d3na00760j)
Supplement: NA-006-D3NA00760J-s001 [file NA-006-D3NA00760J-s001.pdf]

Electronic Supplementary Material (ESI) for Nanoscale Advances.
This journal is © The Royal Society of Chemistry 2023

Design and synthesis of nano-biomaterials based on graphene and local delivery of Cerebrolysin into the injured spinal cord of mouse, promising neural restoration

Ayda Yari-Ilkhchi, [a, b,c] Mehrdad Mahkam, [a*] Abbas Ebrahimi-Kalan, [b] Hamid Soltani Zangbar [b]

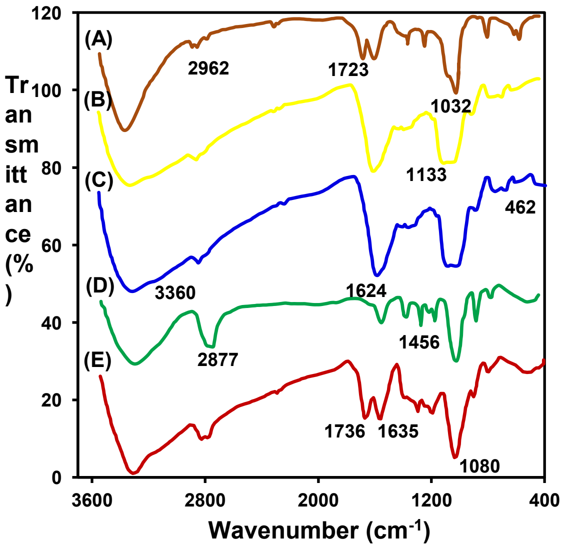

Fig S1. FTIR spectra of (A) GO, (B) GO-SiO_2-NH_2, (C) GO-Au, (D) PEG, and (E) GO-PG.

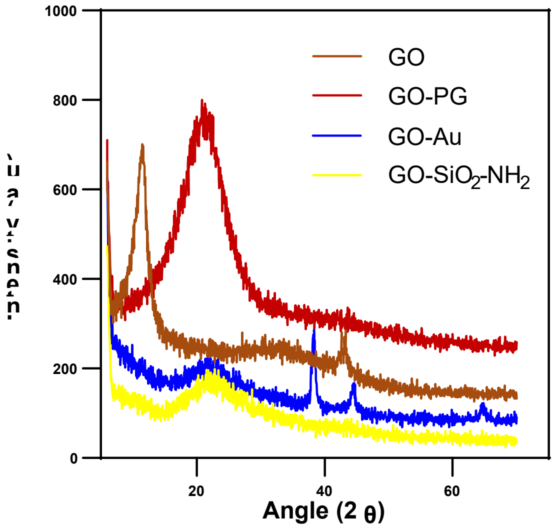

Fig S2. XRD diffractogram of GO, GO-PG, GO-Au, and GO-SiO_2-NH_2.

(a)

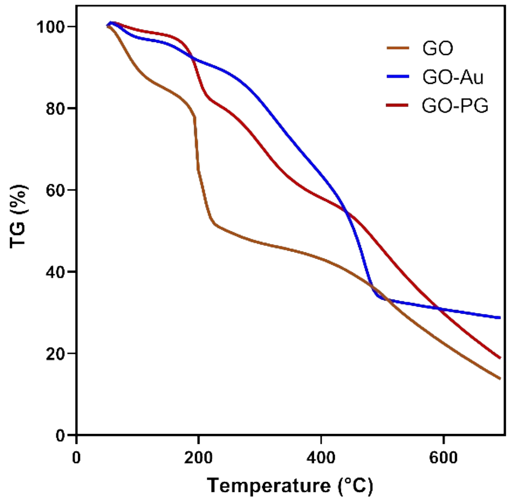

(b)

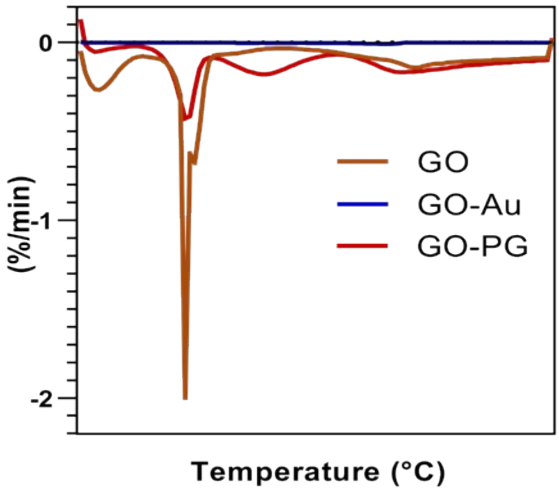

Fig S3. (a) TGA pattern, and (b) DTG curves of GO, GO-PG, and GO-Au.

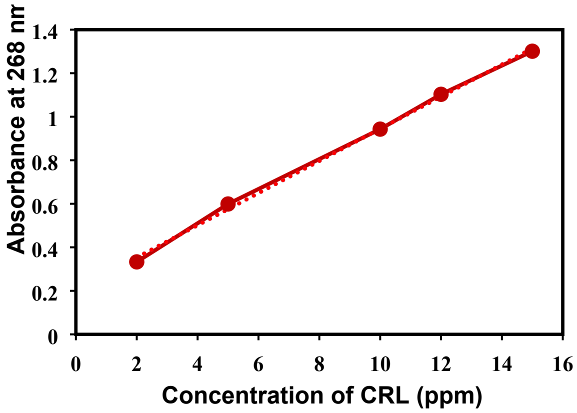

Fig S4. Calibration standard of CRL
